# Could Gas6/TAM Axis Provide Valuable Insights into the Pathogenesis of Systemic Sclerosis?

**DOI:** 10.3390/cimb46070444

**Published:** 2024-07-15

**Authors:** Daria Apostolo, Davide D’Onghia, Alessandra Nerviani, Giulia Maria Ghirardi, Daniele Sola, Mattia Perazzi, Stelvio Tonello, Donato Colangelo, Pier Paolo Sainaghi, Mattia Bellan

**Affiliations:** 1 Department of Translational Medicine, University of Piemonte Orientale (UPO), 28100 Novara, Italy; daria.apostolo@uniupo.it (D.A.); davide.donghia@uniupo.it (D.D.); daniele.sola@med.uniupo.it (D.S.); 20007388@studenti.uniupo.it (M.P.); stelvio.tonello@med.uniupo.it (S.T.); pierpaolo.sainaghi@med.uniupo.it (P.P.S.); mattia.bellan@med.uniupo.it (M.B.); 2 Centre for Experimental Medicine and Rheumatology, Barts and The London School of Medicine and Dentistry, William Harvey Research Institute, Queen Mary University of London, London E1 4NS, UK; g.m.ghirardi@qmul.ac.uk; 3 IRCCS Istituto Auxologico Italiano, UO General Medicine, 28824 Oggebbio, Italy; 4 Internal Medicine and Rheumatology Unit, A.O.U. Maggiore della Carità, 28100 Novara, Italy; 5 Department of Health Sciences, Pharmacology, University of Piemonte Orientale (UPO), 28100 Novara, Italy; donato.colangelo@med.uniupo.it; 6 Center on Autoimmune and Allergic Diseases (CAAD), University of Piemonte Orientale, 28100 Novara, Italy

**Keywords:** Gas6, TAM receptors, systemic sclerosis, biomarkers

## Abstract

Systemic sclerosis (SSc) is a connective tissue disorder characterized by microvascular injury, extracellular matrix deposition, autoimmunity, inflammation, and fibrosis. The clinical complexity and high heterogeneity of the disease make the discovery of potential therapeutic targets difficult. However, the recent progress in the comprehension of its pathogenesis is encouraging. Growth Arrest-Specific 6 (Gas6) and Tyro3, Axl, and MerTK (TAM) receptors are involved in multiple biological processes, including modulation of the immune response, phagocytosis, apoptosis, fibrosis, inflammation, cancer development, and autoimmune disorders. In the present manuscript, we review the current evidence regarding SSc pathogenesis and the role of the Gas6/TAM system in several human diseases, suggesting its likely contribution in SSc and highlighting areas where further research is necessary to fully comprehend the role of TAM receptors in this condition. Indeed, understanding the involvement of TAM receptors in SSc, which is currently unknown, could provide valuable insights for novel potential therapeutic targets.

## 1. Introduction

Systemic sclerosis (SSc) is a rare chronic autoimmune disease preferentially affecting females and subjects with a familiar history [1]. While the prevalence of systemic sclerosis is relatively low, it is associated with higher mortality rates in comparison to other rheumatic diseases [2,3]. SSc can manifest in various ways, and the severity and progression of its symptoms can vary widely among patients [4]. SSc is distinguished by intricate and diverse pathogenic pathways, including vasculopathy, aberrant immune activation leading to the production of autoantibodies, and fibrosis [5]. A deeper understanding of the possible actors contributing to the physiopathology of SSc and their role in this condition is crucial.

Historically, Growth Arrest-Specific 6 (Gas6) and its receptor family Tyro-3, Axl, and MerTK (TAM) have been linked to homeostatic processes in the immune, reproductive, hematopoietic, vascular, and nervous systems. Alongside with this, TAMs and their ligands were shown to be essential to facilitate the phagocytosis of apoptotic cells in these tissues. Specifically, studies regarding the innate immune system previously described TAMs as pleiotropic due to their multifaceted roles in regulating various physiological and pathological processes [6,7]. Moreover, in vivo models in which all TAM receptors were knocked out presented the development of a wide-spectrum autoimmunity [8]. Consequently, the TAM receptor axis constitutes a promising biological system in the context of SSc.

Hitherto, a growing body of data is present in the literature regarding TAMs, their signaling, and their involvement in numerous pathogenic processes across different conditions. As a result, the inhibition of the TAM axis has been suggested as an encouraging treatment approach in various diseases [9,10].

In this review, we aim to elucidate the pathogenesis of systemic sclerosis and the role of the Gas6/TAM system, emphasizing why this system merits further investigation in this field. We performed a literature search for reviews and original articles evaluating the clinical features and pathogenesis of SSc, as well as the role of the Gas6/TAM system in human diseases. Furthermore, we searched the PubMed, Medline, and Cochrane libraries using the following strings: (Gas6 OR TAM receptors) AND (systemic sclerosis OR scleroderma).

### 1.1. Clinical Manifestation of SSc

According to the pattern of skin involvement, SSc patients can be divided into two phenotypes: diffuse cutaneous SSc (dcSSc) and limited cutaneous SSc (lcSSc). In the latter, fibrosis is limited to the face and the distal areas of the limbs, while dcSSc involves a larger skin area; however, both variants may affect internal organs (such as the lungs, gastrointestinal tract, and kidneys), although this eventuality is more common in dcSSc, which, indeed, is burdened by a worse prognosis [11]. The typical onset sign is Raynaud’s phenomenon, which often precedes the diagnosis of SSc. Raynaud’s is related to a recurrent and transient vasospasm of the small digital arteries, triggered by cold or emotional stress and often accompanied by pain and numbness. Raynaud’s phenomenon is characterized by a triphasic sequence of color change: (a) sudden skin pallor typically affecting the fingers, either partial or total, due to vasoconstriction, (b) cyanotic skin changes, also known as a blue attack, and (c) vascular reperfusion and consequent rewarming [12,13].

### 1.2. Complications of SSc

Cardiopulmonary involvement can significantly impact the prognosis of patients with SSc. Pulmonary arterial hypertension (PAH) is a major complication resulting from the progressive remodeling of the pulmonary vasculature. In particular, endothelial injury and inflammation are identified as common precursors. The inflammation disrupts the balance between vasoactive, proliferative mediators and antiproliferative vasodilators within the endothelium, contributing to pulmonary artery vasoconstriction and cellular proliferation [14]. Concomitantly, inflammatory cell infiltrates composed of macrophages, dendritic cells, and T and B lymphocytes have been found in PAH, implicating the participation of cytokines, including interleukin (IL)-1 and IL-6, and chemokines such as C-X3-C motif chemokine ligand 1 (CX3CL1) in a dysregulated proliferation of pulmonary artery smooth muscle cell (PASMC) and endothelial cell (EC) release of cytokines; the net effect is an increase in pulmonary vascular resistance [15]. Another common manifestation of SSc is interstitial lung disease (ILD); even if the pathophysiology is still unclear, it is believed that the abnormal interplay among ECs, mononuclear cells, and fibroblasts stimulates profibrotic cytokines release in the context of vascular hyperreactivity and tissue hypoxia, eventually driving the aberrant deposition of the extracellular matrix (ECM) in lung parenchyma [16]. In addition to this, gastrointestinal tract, central and peripheral nervous system, musculoskeletal system, and kidney involvement contribute to the high rate of mortality in SSc patients [17,18]. The current treatment options are mainly based on immunosuppressive agents, although, recently, an antifibrotic drug (nintedanib) was shown to be effective for the management of lung disease. A more intensive therapeutic option involves autologous hematopoietic stem cell transplantation for patients with rapidly progressive dcSSc [19].

### 1.3. Pathogenesis of SSc

#### 1.3.1. Vascular Injury

**Figure 1:**
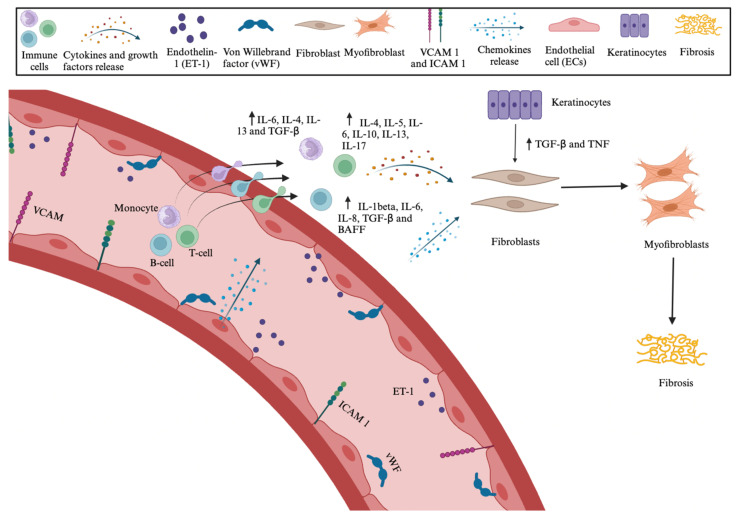
Pathogenetic mechanisms involved in SSc. Endothelial cells are activated upon vascular damage with the consequent expression of adhesion molecules and production of vWF, ET-1, and chemokines. The injured endothelium produces molecules capable of recruiting immune cells. Inflammatory infiltrates composed of monocytes, macrophages, and T cells and B cells sustain the release of proinflammatory and profibrotic cytokines. Resident fibroblasts undergo a phenotypic conversion to myofibroblasts, the effector cells of fibrosis. Abbreviation: vascular cell adhesion molecule-1, VCAM-1; intercellular cell adhesion molecule-1, ICAM-1; endothelin-1, ET-1; B cell activating factor, BAFF; interleukin, IL; tumor necrosis factor, TNF; Transforming Growth Factor-β, TGF-β; Von Willebrand factor, vWF; endothelial cell, ECs.

Although the pathophysiology of SSc is not completely elucidated, it is known to be characterized by microvascular injury, the production of autoantibodies, and fibroblast dysfunction, which induces an increased deposition of ECM (Figure 1) [17,20]. Indeed, vascular damage is an early event in SSc that leads to a cascade of processes contributing to the disease development. Infectious agents, cytotoxic T cell nitric oxide (NO)-related free radicals, and oxidative stress have been recognized as potential actors in EC damage, even if the exact role of each of these stressors is not fully understood [21,22,23]. The damaged ECs release endothelin-1 (ET-1) and von Willebrand factor (vWF); as a result, the homeostatic balance between vasodilation and vasoconstriction is deranged, and the increase in vessel tone contributes to tissue hypoxia [24,25,26]. In response to the vascular damage, ECs also increase the expression of adhesion molecules (vascular cell adhesion molecule-1, VCAM-1; intercellular cell adhesion molecule-1, ICAM-1; E-selectin) and chemokines (C-C motif chemokine ligand (CCL) 2; CCL3; IL-8; CCL18) [27,28].

#### 1.3.2. Inflammation

Innate and adaptive immune cells, particularly monocytes, macrophages, dendritic cells, and subsets of T cells and B cells, also contribute to the inflammation underlying this condition [29,30]; indeed, the inflammatory infiltrates engage with resident fibroblasts, releasing profibrotic and proinflammatory cytokines [20,31,32]. In addition, keratinocytes and dermal fibroblasts reciprocally release signaling molecules or factors (IL-1 produced by keratinocytes and keratinocyte growth factor by fibroblasts), contributing to the homeostasis [33]. In SSc, this crosslink is disrupted, and the final result is an upregulation of Transforming Growth Factor-β (TGF-β) signaling and the release of peptides with Damage-Associated Molecular Pattern (DAMP) properties, along with tumor necrosis factor (TNF), which perpetuate the inflammation [34].

#### 1.3.3. Activation of Fibroblasts

Ultimately, resident fibroblasts, stimulated by the profibrotic inflammatory environment, proliferate and differentiate into myofibroblasts, mainly responsible for the excessive deposition of the extracellular matrix, which is mostly composed of highly crosslinked type I collagen [35,36]. Myofibroblasts are specialized fibroblasts that display prominent cytoplasmatic stress fibers, α-smooth muscle actin (α-SMA), and express receptors associated with profibrotic pathways, including TGF-β [37]. SSc fibroblasts also express platelet-derived growth factor (PDGF) and its receptors; PDGF is a chemoattractant for fibroblasts, forcing them to produce collagen and to secrete TGF-β1, IL-6, and monocyte chemoattractant protein 1 (MCP-1), thus strengthening the development of fibrosis [38]. Fibrosis is a common response to chronic inflammation and injury; it is characterized by the disruption of structure and function of the affected tissues. In fibrotic tissues, the excess of EMC dominates the tissues, leading to a more acellular composition and tissue stiffness that compromise the normal flexibility, collectively resulting in the loss of functional integrity [39,40].

#### 1.3.4. Autoantibodies in SSc

The search for autoantibodies is particularly useful for both diagnosis and prognostic stratification. Indeed, SSc-associated autoantibodies are clinically important for the diagnosis and the prediction of organ involvement, since they are disease-specific antibodies that give indications on clinical features, severity, and prognosis [41]. SSc autoantibodies are mutually exclusive, and recent evidence proposes that they could help guide treatment strategies [42]. A wide range of autoantibodies is involved in SSc, and antinuclear antibodies (ANA) are usually detected in more than 90% of patients [43]. The most common antinuclear antibodies capable of defining well-described cardiopulmonary complications are the anti-centromere antibodies (ACA), anti-DNA topoisomerase I antibodies (anti-topo I), anti-RNA polymerase III antibodies (anti-RNA pol III), and anti-Th/To antibodies (anti-Th/To) (Table 1) [44,45,46,47].

Other autoantibodies may be indicative of an overlap syndrome, as they are not specific for systemic sclerosis, but rather, they are also found in other rheumatic diseases (e.g., Sjögren’s syndrome, systemic lupus erythematosus, rheumatoid arthritis, polymyositis, and dermatomyositis) [48,49,50]. These non-specific autoantibodies include anti-U3-RNP antibodies, anti-U1-RNP antibodies, anti-U11/U12-RNP antibodies, anti-Ku antibodies, anti-PM–Scl antibodies, and anti-Ro antibodies [44,48].

## 2. Gas6/TAM System

### 2.1. Gas6/TAM System’s Functions

**Figure 2:**
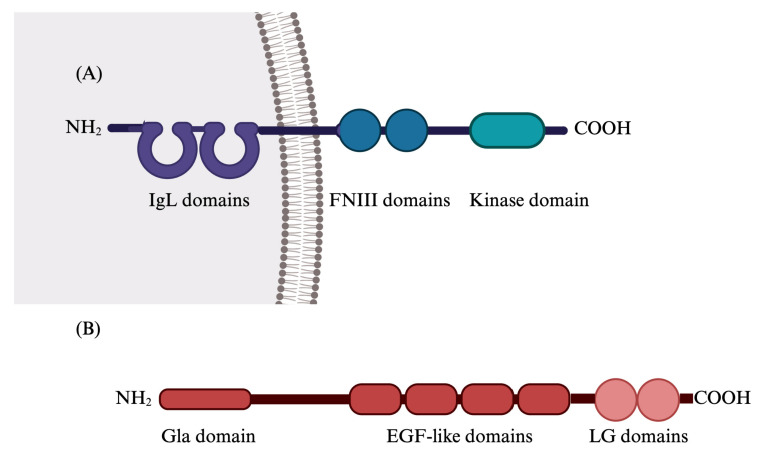
Structure of TAMs (**A**). TAMs consist of immunoglobulin-like (IgL) domains, two fibronectin domains, and a kinase domain. Structure of Gas6 (**B**). Gas6 consists of a gamma-carboxyglutamic acid (Gla) domain, four epidermal growth factor (EGF)-like domains, and two laminin G (LG)-like domains. Created with https://www.BioRender.com (accessed on 18 June 2024).

The Gas6/TAM system is a highly pleiotropic system, considered one of the main actors in the context of inflammation, vascular integrity, and homeostasis. TAM is the acronym of Tyro3, Axl, and MerTK, a group of tyrosine kinase receptors [6]. They are structurally similar and include two extracellular fibronectin type III, two immunoglobulin (Ig)-like domains, and one kinase domain, with a signature motif (KWIAIES) specific for TAM receptors [51]. The main ligands for TAM receptors are vitamin K-dependent proteins, namely Gas6 and Protein S (Pros1) [52,53,54,55]. Gas6 and Protein S share approximately 43% amino acid sequence homology and have the same domain structure; unlike Gas6, Pros1 is only able to bind Tyro-3 and MerTK but not Axl. Structurally, Gas6 and Pros1 comprehend a γ-carboxyglutamate (Gla)-rich domain, four epidermal growth factor-like domains, and one sex hormone-binding globulin (SHBG)-like domain that contains two laminin G-like domains (Figure 2) [56]. Following the binding of the ligand, the receptor dimerizes, allowing the trans-autophosphorylation of the intracellular tyrosine kinase domains and downstream signal transmission, which generally follows the MEK/ERK, PI3K/AKT, and JAK/STAT pathways [57,58]. Axl and MerTK receptors can also be cleaved by A Disintegrin and metalloproteinase domain-containing protein 10 (ADAM10) and A Disintegrin and metalloproteinase domain-containing protein 17 (ADAM17), and their soluble forms (sAxl and sMer) are still able to bind Gas6 protein, thus regulating its function [59,60]. In spite of the structural similarity, Pros 1 is primarily expressed in the liver and endothelial cells, while Gas6 is widely expressed in vascular smooth muscle cells, kidneys, lungs, intestine, heart, and monocytes [61].

The Gas6/TAM system is involved in many biological processes, including cellular homeostasis, vascular integrity, platelet function, regulation of inflammatory responses, adhesion and migration, phagocytosis, and apoptosis regulation, as well as fibrotic evolution [62,63,64,65,66,67]. As a regulator of the inflammatory response, the TAM system works to obviate the chronic activation of antigen-presenting cells (APCs) by attenuating the inflammatory pathways. Indeed, innate immune cells, including macrophages and dendritic cells, recognize pathogens through pattern recognition receptors (PRRs), such as Toll-like receptors (TLRs). This triggers a huge release of cytokines and chemokines, contributing to local inflammation [68,69]. TAM receptors are able to upregulate the suppressors of cytokine secretion (SOCS) proteins, particularly SOCS1 and SOCS3. TAM receptor-dependent activation by Gas6 ends up in TLR2 and TLR6 inhibition and SOCS1 and SOCS3 induction, hence dampening the inflammatory response [7,70]. In mouse macrophages, Gas6 and Pros1 have been demonstrated to synergistically suppress both the basal and the TLR-triggered production of inflammatory cytokines, including IL-6, TNF-α, and IL-1β, through the activation of TAM receptors [71]. Moreover, the recognition of TAM receptors by Gas6 is fundamental for the transition from inflammation to its resolution, with tissue repair and healing [72]. TAM receptors and their ligands Gas6 and Pros1 are considered crucial regulators of efferocytosis, acting as bridging molecules that facilitate the recognition and engulfment of apoptotic cells by macrophages [73,74]. This process maintains tissue homeostasis and prevents the development of chronic diseases and autoimmunity. Indeed, impairment of efferocytosis with the defective removal of apoptotic bodies and the subsequent failure of inflammation resolution result in the development of various chronic inflammatory and autoimmune diseases [75,76,77].

Furthermore, the vasculature could represent a target for the Gas6/Axl axis, as both Gas6 and Axl molecules are expressed by various cell types within blood vessels, including ECs, pericytes, and smooth muscle cells [78,79,80]. After injury to the blood vessel wall, VSMCs undergo proliferation and exhibit migratory behavior. This dynamic and highly ordered program involves the expression of various molecules, including growth factors, receptors, and intracellular mediators [81]. Following injury, overexpression of Axl has been found in neointima VSMCs of rat carotid arteries, suggesting its role as a potential contributor to VSMC proliferation, its expression regulated by angiotensin II [80]. Moreover, Gas6/Axl interaction is able to activate downstream survival of the PI3K-Akt pathway, acting as an antiapoptotic mechanism preventing VSMC apoptosis, in turn contributing to the response to vascular injury [66]. In addition, an in vitro study on human umbilical vein endothelial cells (HUVECs) demonstrated that recombinant human Gas6 is protective against apoptotic stimuli, and Gas6-Axl activation fosters EC survival via Akt phosphorylation and NF-κB activation [82]. Moreover, the binding between TAM receptors and Gas6 amplifies EC activation, resulting in an enhanced expression of the adhesion molecules VCAM-1 and ICAM-1 [83]. Mouse models of inflammation (e.g., heart transplantation, endotoxemia, and vasculitis) have demonstrated that Gas6 contributes to leukocytes and platelets sequestration on activated endothelium and supports leukocyte extravasation and inflammation [83].

Gas6 has also been demonstrated to participate in wound repair through its signaling pathways involving Mer, RhoA, and downstream effectors. In particular, it stimulates the production of epithelial growth factor hepatic growth factor (HGF) in macrophages (RAW 264.7 cells), which, in turn, boosts the proliferation of epithelial cells, promoting wound repair. More specifically, the activation of the Gas6/MerTK downstream signaling pathway composed of RhoA/protein kinase B (Akt)/mitogen-activated protein (MAP) kinases, including p38 MAP kinase, extracellular signal-regulated protein kinase, and Jun NH2-terminal kinase, results in the upregulation of HGF at both the mRNA and protein levels [84]. It has also been demonstrated that the Gas6/Axl or MerTK signaling pathway could prevent the TGF-β1-induced epithelial to mesenchymal transition (EMT) and exert an inhibitory effect on the migration and invasion in alveolar type II (ATII) ECs. Gas6 can block non-Smad TGF-β1 signaling and downregulates the mRNA expression of the transcription factors associated with EMT. Moreover, in vitro, Gas6 stimulation enhances COX-2 expression and the subsequent secretion of prostaglandin E2 (PGE2) and prostaglandin D2 (PGD2), mediating anti-EMT effects in an autocrine/paracrine manner [85].

Ultimately, Gas6 appears to have a dual role in tissues, wherein its protective effect on inflammation is juxtaposed with profibrotic properties [86]. Indeed, while TAM receptors can have anti-inflammatory effects in certain contexts, persistent activation may lead to a chronic inflammatory state, contributing to the perpetuation of fibrosis [87,88,89,90,91]. Activation of these receptors by Gas6 can trigger signaling pathways that influence fibrogenesis [92,93]. A recent study demonstrated that Gas6 is able to upregulate TGF-β, which is also a central regulator of fibrosis that promotes the synthesis of extracellular matrix components, such as collagen, leading to tissue fibrosis [94,95].

### 2.2. Gas6/TAM System in Human Diseases

#### 2.2.1. Gas6/TAM in Cancer

The Gas6/TAM system has been widely studied in different human conditions, with a particular focus on cancer [9,96,97,98,99,100,101,102]. The tumor microenvironment is rich in phosphatidylserine (PtdSer), provided from several sources (e.g., intra-tumoral apoptotic cells and tumor-derived exosomes) and exposed on the membranes by viable cancer cells. The interaction between TAMs on APCs and PtdSer on cancer cells through either Gas6 or Pros1 attenuates the immune response, leading to survival signals and favoring an immunosuppressive and anti-inflammatory microenvironment [103,104,105]. Apart from PtdSer, the Gas6/TAM system has been associated with prolonged survival and increased proliferation of cancer cells, as well as with the regulation of cancer cells migration and invasion [106,107]. In particular, the overactivation of TAMs on tumor cells results in the activation of various downstream oncogenic pathways, including MEK/ERK, PI3K/AKT, JAK/STAT, and p38, promoting cell growth and proliferation. The TAM system regulates migration and invasion through RHO, matrix metalloproteinase 9 (MMP9), and focal adhesion kinase 1 (FAK1), promoting metastasis formation [108,109]. The overexpression of TAMs, particularly Axl, is involved in various solid and hematological tumors, and generally results in higher metastatic risk and worse prognosis [110,111]. Targeting Gas6 or TAM receptors has emerged as a potential therapeutic strategy in cancer treatment; indeed, inhibiting the Gas6/TAM axis could potentially disrupt the pro-tumorigenic signaling pathways and enhance the effectiveness of anticancer therapies [112,113,114,115]. In the context of targeting the Gas6/TAM axis, some studies have focused on developing inhibitors that target the TAM receptors rather than Gas6 itself [107]. Therapies targeting MerTK, including small molecule inhibitors and blocking antibodies, have demonstrated the efficacy of MerTK inhibitors in specific MerTK-sensitive tumors. Small molecule inhibitors with specificity for MerTK/FLT3, such as MRX2843 and UNC1666, have shown a substantial impact on cell growth in acute myeloid leukemia (AML); acute lymphoblastic leukemia (ALL); and melanoma in various experimental settings, including cell lines, murine models, and primary patient tumor samples [116,117]. Regarding Axl-targeted drugs, monoclonal antibodies specific to Axl, such as YW327.6S2 and 20G7-D9, have demonstrated their ability to inhibit the growth of cancer cells [118]. Many clinical trials focus on targeting Axl with small molecule selective inhibitors (BGB324 and TP-0903), antibody–drug conjugates (BA3011), anti-Axl Fc fusion protein AVB-S6-500, and multitargeted inhibitors (ONO-7475, Merestinib and Sitravatinib). These compounds were employed either alone or in combination with other drugs and showed significant therapeutic effects [119,120].

#### 2.2.2. Gas6/TAM System in Liver Diseases

Besides cancer, Gas6/TAMs are also involved in liver diseases [86,121,122,123,124]. The Gas6/TAM axis might be beneficial in acute liver injury modulating immune responses and promoting tissue repair but does potential harm in chronic liver diseases [125]. Considering the current literature, Gas6 has been reported to have a hepatoprotective role in certain liver pathologies, mainly by dampening inflammatory processes. Indeed, by suppressing the production of proinflammatory cytokines and mediating efferocytosis and APC activity, Gas6 can limit excessive inflammation. Specifically in ischemia/reperfusion-induced damage in mice, Gas6 was demonstrated to protect mouse hepatocytes from hypoxia and reduce the production of inflammatory cytokines [126]. Regarding a wound healing response to liver injury, Gas6 knockout mice showed a delayed resolution of liver necrotic areas, suggesting that Gas6/Axl signaling is essential for regulating the liver inflammation necessary for normal wound healing [127]. On the contrary, this system behaves differently in chronic conditions. The Gas6/MerTK signaling pathway appears to promote fibrosis in vitro through hepatic stellate cell (HSC) activation and the consequent upregulation of several genes involved in fibrosis. Blocking this binding through MerTK inhibitors (e.g., UNC569), MerTK gene silencing, or inhibiting Gas6/TAM activation by RU-301 resulted in decreased fibrosis in in vitro models [90,128,129]. Similarly, Axl knockout mice and Axl pharmacological inhibition via Bemcentinib in mice have been demonstrated to ameliorate liver fibrosis induced by chronic administration of carbon tetrachloride (CCl_4_) [88]. In humans, Axl upregulation stimulates tumor progression in hepatocellular carcinoma (HCC) by influencing cancer cell plasticity and the tumor microenvironment [130].

#### 2.2.3. Gas6/TAM System in Lung Diseases

Furthermore, the Gas6/TAM system contributes to both inflammation and fibrosis, two key events involved in ILD. In patients with Idiopathic Pulmonary Fibrosis (IPF), Gas6, Axl, and Tyro3 were elevated compared to the healthy control, and phosphorylated Axl was higher in rapid progressors versus slow progressors [131]. Moreover, the activation of Axl was observed in the epithelial remodeling of lung fibrosis, possibly resulting in the loss of the epithelial barrier [132]. On the contrary, Gas6/Axl signaling regulates alveolar inflammation in ischemia–reperfusion-induced acute lung injury (IR-ALI) through the upregulation of SOCS3 and downstream pathways, attenuating the injury and the inflammation [133]. Moreover, Gas6/TAM signaling can activate pulmonary fibroblasts, and small molecule inhibitors, such as the Axl inhibitor BGB324, show promise in inhibiting fibroblast activation compared to specific antibodies directed to Gas6 or Axl [131].

#### 2.2.4. Gas6/TAM System in Infectious Diseases

More recently, this system has also been shown to support the immune response in COVID-19 and post-COVID-19 sequelae [62,67]. Indeed, in COVID-19 patients, Gas6 levels increase progressively with the severity of the disease and predict adverse outcomes [134,135]; a derangement of this system has also been associated with hair loss one year after hospital discharge due to COVID-19 [136]. Moreover, Axl has been proposed as a candidate receptor for severe acute respiratory syndrome coronavirus 2 (SARS-CoV-2) in view of its role in promoting the viral infection of pulmonary and bronchial epithelial cells. Axl has been identified to specifically interact with the spike protein of SARS-CoV-2, the N-terminal domain. Overexpression of Axl and angiotensin-converting enzyme 2 (ACE2) receptor, known as the primary cellular receptor for SARS-CoV-2, in HEK293T cells has been demonstrated to be equally efficient in promoting viral entry. Axl deficiency significantly reduced the viral infection in H1299 pulmonary cells and human primary lung epithelial cells [137]. Axl inhibition has been also proposed as a potential alternative for COVID-19 treatment due to its reported antiviral activities in preclinical studies [138,139].

Apart from SARS-CoV-2, Ebola and Vaccinia viruses can manipulate host cells to aid cell entry and enhance infection through TAM receptors. Specifically, Gas6, by binding to PtdSer on the surface of the virion, links the virus to the membrane of macrophages and other phagocytes through interactions with TAM receptors, thus facilitating viral internalization [140,141,142]. Concerning ZIKA virus (ZIKV), the interaction among ZIKV-Gas6-Axl leads to the downregulation of various interferons and proinflammatory cytokines, thereby suppressing innate immune and inflammatory responses [143].

#### 2.2.5. Gas6/TAM System in Cardiovascular Diseases

Emerging evidence indicates that the Gas6/TAM system may be involved in heart failure (HF), with elevated levels of Axl observed in both myocardial expression and serum concentration among HF patients compared to control groups [144]. Moreover, increased plasma Gas6 levels were linked to a higher risk of both all-cause and cardiovascular mortality in patients with acute heart failure (AHF) [145]. A notable decrease in long non-coding RNA GAS6-AS1 expression was noted in the plasma of patients with acute myocardial infarction (AMI) compared to individuals without heart conditions [146]. Regarding MerTK, upon analyzing sections of carotid artery endarterectomy samples, a positive correlation between sMer and the percentage of necrosis has been observed. Notably, sMer was not detected in nonatherosclerotic human arteries. Furthermore, plaques from symptomatic patients displayed increased levels of sMer compared to those from asymptomatic patients [147]. Enhancing MerTK function or preventing its cleavage could be promising therapeutic approaches for cardiovascular disease. Concerning Tyro3, it could have a protective role by suppressing type 2 immune responses that promote cardiac fibrosis, but further investigation could reveal potential for Tyro3 as a therapeutic target to prevent fibrosis post-cardiac injury [148].

#### 2.2.6. Gas6/TAM System in Rheumatic Diseases

In the context of rheumatic and autoimmune diseases, the serum levels of sTyro3 are increased in patients with rheumatoid arthritis (RA) and correlate directly with disease activity and bone destruction [149]. Gas6 has been reported to be present in the synovial tissue of patients with RA, with lower levels observed in erosive RA compared to non-erosive RA [150]. More recently, AXL and MERTK RNA expression levels were measured in RA patients from the R4RA clinical trial and treated with a second-line biologic agent, either rituximab or tocilizumab [151]. Upon blocking the IL-6 pathway downstream of treatment with the IL-6 receptor inhibitor tocilizumab, the Axl synovial transcript levels were found significantly upregulated, possibly indicating that modulation of the IL-6 pathway can influence Axl expression [152].

In osteoarthritis, the interaction of Gas6 with the Axl receptor exhibits anti-inflammatory effects associated with the upregulation of SOCS1/3 in fibroblast-like synoviocyte and biopsies from the joints of patients [153]. Moreover, the overexpression of *Pros1* or *Gas6* genes successfully reduced arthritis pathology in a murine model of collagen-induced arthritis [154].

In patients with primary Sjögren’s syndrome (pSS), plasma levels of sMer were elevated and associated with disease activity and inflammatory response [155]. In systemic lupus erythematosus (SLE) patients, a derangement of the Gas6/TAM system with increased Gas6, sAxl, and sMer plasma concentrations has been correlated with disease activity [156,157] and with the parameters of renal involvement in SLE patients with lupus nephritis (LN) [158].

### 2.3. Gas6/TAM System in Systemic Sclerosis

Given the above, it is reasonable to postulate that the Gas6/TAM system could also be involved in the pathogenesis of SSc. The rationale for implicating the Gas6/TAM axis in SSc derives from the proven involvement of the system in vascular integrity and its overexpression in vascular injury, the role of Gas6/TAMs in efferocytosis, which is a fundamental mechanism for the prevention of autoimmunity, and the interplay of Gas6/TAMs in inflammation and fibrosis. Indeed, this biological system, as previously described, dampens the inflammatory response, driving towards the profibrogenic route. As detailed above, these are all well-known events implicated in the pathogenesis of SSc.

To date, however, papers assessing the potential diagnostic and pathogenetic roles of Gas6 and TAMs in SSc are lacking. Our group evaluated a cohort of 125 SSc (or SSc overlap) patients; out of them, 19 (15%) were affected by pulmonary hypertension (PH), while 39 (31%) had a certain degree of ILD, which was characterized by a severe functional impairment in 6 (5%) of them. We assessed whether the circulating levels of Gas6, sAxl, and sMer marked the presence of cardiopulmonary involvement. Interestingly, we reported that the sMer plasma concentration was significantly higher in the 14 patients with PAH associated with connective tissue disorders (CTD-PAH) compared to those measured in the patients without PAH or affected by PH not related to CTD. In this context, a sMer increase may result from a combination of a dysfunctional endothelium and an impairment of the mechanisms that dampen inflammation in the pulmonary artery vessel wall [159].

In addition to this, the circulating Gas6 and sAxl plasma levels were slightly increased in mild ILD patients compared to patients without ILD, possibly reflecting this system as either a marker of progression to fibrosis or an indicator of a dysregulated control of inflammation [159]. This finding is particularly interesting since, as previously described, the system has been claimed as a promising target in the management of IPF. IPF shares similarities with CTD-ILD and, recently, an antifibrotic agent, namely nintedanib, was shown to be effective both in IPF and progressive CTD-ILD. Therefore, it is reasonable that targeting fibrosis through Axl inhibition might be equally effective in both conditions.

However, following the scarcity of research papers dealing with this topic, the interpretation of these findings is merely speculative; we believe that this promising basis should support further investigation about the role of Gas6/TAMs in the pathogenesis of SSc.

## 3. Treatment of SSc

Although direct effects on TAM receptors are not well documented, the broad immunosuppressive properties of drugs used to treat SSc might indirectly impact TAM receptor signaling by modulating the immune environment.

Regarding biologic disease-modifying antirheumatic drugs (bDMARDs), IL-6 inhibition by tocilizumab resulted in an increased expression of Axl and MerTK in the RA synovial tissue. This modulation was associated with a reduction in synovial inflammation, suggesting that IL-6 inhibition may exert anti-inflammatory effects partly through the upregulation of TAM receptors. The same trend was not observed after the depletion of B cells in the rituximab-treated RA patient group [152].

Commonly used for autoimmune diseases, glucocorticoids can induce ProS-dependent phagocytosis of apoptotic cells by macrophages through the upregulation of MerTK expression [160,161], thus implicating its potential role in TAM receptor signaling.

Nintedanib is a tyrosine kinase inhibitor that has been recently approved as an antifibrotic agent for the treatment of SSc-ILD [162], and considering that TAM receptors belong to the tyrosine kinase family, it is plausible that they could play a role in the pathophysiology of SSc and its treatment.

As with all other treatments used in SSc, there is no direct evidence of the involvement of the Gas6/TAM system.

While direct evidence of the effects of these treatments on the Gas6/TAM system is sparse, its role in reducing inflammation and cytokine levels suggests that they may indirectly modulate this system (Table 2).

## 4. Conclusions

Despite advances in understanding the underlying mechanisms and clinical manifestations of SSc, there are aspects that have not been adequately explored. The discovery of new therapeutic targets and specific diagnostic and prognostic markers is crucial for optimizing the management of systemic sclerosis and its life-threatening complications. Regardless of the limited data on the Gas6/TAM system in the context of SSc, this review presents possible contributions of this system to the pathogenesis of the condition. Indeed, the Gas6/TAM system has been implicated in maintaining vascular integrity, being overexpressed in vascular damage. It also plays a role in efferocytosis, a crucial mechanism for preventing autoimmunity, and it mitigates the inflammatory response, guiding toward a profibrogenic pathway. So far, these aspects, although characterized in other conditions, have not been investigated in SSc. To this day SSc treatment is confined to immune modulation and antifibrotic therapy, making the discovering of new targets and the exploration of new therapeutic approaches vital, as they would fill a huge unmet need.

Targeting of TAM receptors has recently been further explored spanning numerous diseases and constitutes a promising field of translational research. A deep understanding of TAM involvement in SSc could pave the way for unveiling their potential as therapeutic targets in this condition. To conclude, it would be worthwhile to explore this system deeper, as it may provide valuable insights into the pathogenesis of systemic sclerosis.

## Figures and Tables

**Table 1:** Association between autoantibodies in SSc and clinical manifestations. Abbreviations: anti-centromere antibodies, ACA; anti-DNA topoisomerase I antibodies, anti-topo I; anti-RNA polymerase III antibodies, anti-RNA pol III; limited cutaneous systemic sclerosis, lcSSc; diffuse cutaneous systemic sclerosis, dcSSc; pulmonary arterial hypertension, PAH; interstitial lung disease, ILD.

Autoantibodies	Phenotypes	Target	Clinical Associations
ACA	lcSSc	ACA are mainly directed towards three centromere proteins, namely CENP-A, B, and C.	Cutaneous calcinosis, dermal thickness of hands and/or feet distally from elbow and knee, respectively, and PAH.
Anti-topo I	dcSSc	Anti-topo I are directed towards a nuclear protein of 70–100 kD, clustered with DNA molecules and involved in altering DNA chain conformation during cellular replication.	Ischemic digital ulcers, flexion contractures in metacarpophalangeal and proximal interphalangeal joints, hand disability, and progressive pulmonary fibrosis.
Anti-RNA pol III	dcSSc	Anti-RNA pol III antibodies are reactive with RNA polymerase III.	Joint contractures, scleroderma renal crisis
Anti-Th/To	lcSSc	Anti-Th/To are directed towards protein components of the RNase MRP complex.	ILD and pericarditis.

**Table 2:** This table provides an overview of the effects of various treatments on the Gas6/TAM system, highlighting their mechanisms of action, evidence of involvement, or potential impacts on TAM receptor activity not experimentally proven yet. Abbreviations: tocilizumab, TOC; rituximab, RTX; biologic disease-modifying antirheumatic drugs, bDMARDs.

Treatments	Effects	Involvement in Other Conditions	Possible Involvement of Gas6/TAM Axis	References
TOC and RTX, bDMARDs	TOC: Inhibition of the IL-6-mediated signaling pathways, leading to a reduction in inflammation and immune response modulation.RTX: Depletion of B cells.	Proven.	Increased expression of Axl and MerTK in the RA synovial tissue, suggesting that IL-6 inhibition may exert part of its anti-inflammatory effects through upregulation of TAM receptors.	[152]
Prednisolone, glucocorticoids	Anti-inflammatory and immunosuppressive properties.	Proven.	Glucocorticoids can upregulate the expression of MerTK enhancing the clearance of apoptotic cells and promoting anti-inflammatory pathway.	[160,161]
Nintedanib, tyrosine kinase inhibitor	It targets multiple tyrosine kinases involved in the processes of fibrosis, inflammation, and vascular remodeling.	Proven.	Gas6/TAM receptor activity contributes to the activation of pulmonary fibroblasts in IPF and targeting of TAM receptors alleviates fibrotic mechanisms.	[131]

## Data Availability

Not applicable.
